# The Fiber Knob Protein of Human Adenovirus Type 49 Mediates Highly Efficient and Promiscuous Infection of Cancer Cell Lines Using a Novel Cell Entry Mechanism

**DOI:** 10.1128/JVI.01849-20

**Published:** 2021-01-28

**Authors:** Alexander T. Baker, James A. Davies, Emily A. Bates, Elise Moses, Rosie M. Mundy, Gareth Marlow, David K. Cole, Carly M. Bliss, Pierre J. Rizkallah, Alan L. Parker

**Affiliations:** a Division of Cancer and Genetics, School of Medicine, Cardiff University, Cardiff, United Kingdom; b Division of Infection and Immunity, School of Medicine, Cardiff University, Cardiff, United Kingdom; International Centre for Genetic Engineering and Biotechnology

**Keywords:** adenoviruses, surface receptor, oncolytic viruses, anticancer therapy

## Abstract

The human adenovirus (HAdV) phylogenetic tree is diverse, divided across seven species and comprising over 100 individual types. Species D HAdV are rarely isolated with low rates of preexisting immunity, making them appealing for therapeutic applications. Several species D vectors have been developed as vaccines against infectious diseases, where they induce robust immunity in preclinical models and early phase clinical trials. However, many aspects of the basic virology of species D HAdV, including their basic receptor usage and means of cell entry, remain understudied. Here, we investigated HAdV-D49, which previously has been studied for vaccine and vascular gene transfer applications. We generated a pseudotyped HAdV-C5 presenting the HAdV-D49 fiber knob protein (HAdV-C5/D49K). This pseudotyped vector was efficient at infecting cells devoid of all known HAdV receptors, indicating HAdV-D49 uses an unidentified cellular receptor. Conversely, a pseudotyped vector presenting the fiber knob protein of the closely related HAdV-D30 (HAdV-C5/D30K), differing in four amino acids from HAdV-D49, failed to demonstrate the same tropism. These four amino acid changes resulted in a change in isoelectric point of the knob protein, with HAdV-D49K possessing a basic apical region compared to a more acidic region in HAdV-D30K. Structurally and biologically we demonstrate that HAdV-D49 knob protein is unable to engage CD46, while potential interaction with coxsackievirus and adenovirus receptor (CAR) is extremely limited by extension of the DG loop. HAdV-C5/49K efficiently transduced cancer cell lines of pancreatic, breast, lung, esophageal, and ovarian origin, indicating it may have potential for oncolytic virotherapy applications, especially for difficult to transduce tumor types.

**IMPORTANCE** Adenoviruses are powerful tools experimentally and clinically. To maximize efficacy, the development of serotypes with low preexisting levels of immunity in the population is desirable. Consequently, attention has focused on those derived from species D, which have proven robust vaccine platforms. This widespread usage is despite limited knowledge in their basic biology and cellular tropism. We investigated the tropism of HAdV-D49, demonstrating that it uses a novel cell entry mechanism that bypasses all known HAdV receptors. We demonstrate, biologically, that a pseudotyped HAdV-C5/D49K vector efficiently transduces a wide range of cell lines, including those presenting no known adenovirus receptor. Structural investigation suggests that this broad tropism is the result of a highly basic electrostatic surface potential, since a homologous pseudotyped vector with a more acidic surface potential, HAdV-C5/D30K, does not display a similar pantropism. Therefore, HAdV-C5/D49K may form a powerful vector for therapeutic applications capable of infecting difficult to transduce cells.

## Abstract (precis)

Adenoviruses are powerful tools experimentally and clinically. To maximize efficacy, the development of serotypes with low preexisting levels of immunity in the population is desirable.

## INTRODUCTION

Human adenoviruses are divided into seven species, A to G (1). These nonenveloped, icosahedral viruses have garnered significant interest as therapeutic vectors since they can be grown and purified to high titers and because the double-stranded DNA genome is readily amenable to genetic modification, enabling the overexpression of therapeutic transgenes (2, 3). Similar techniques can also be applied to genetically alter the virus structural genes, creating modified viral tropisms which are retained by progeny virions after replication.

Clinically, adenoviruses have been developed as vectors for gene therapy, as vaccines, and as oncolytic virotherapies (1, 4, 5). However, efficacy in these applications can be hampered by preexisting immunity against the therapeutic vector in the population resulting from prior exposure to the wild-type pathogen. Such preexisting immunity is likely to reduce the therapeutic index of such systems, due to rapid and efficient removal of the engineered therapeutic agent by the reticuloendothelial system (6, 7). This is especially relevant where the therapeutic is based on the most commonly studied species C adenovirus, human adenovirus type 5 (HAdV-C5), where neutralizing antibodies are found in ∼90% of patients from sub-Saharan Africa and ∼30% of a Scottish patient cohort (8, 9).

A promising means to circumvent preexisting immunity is through the development of viruses with naturally low seroprevalence rates as therapeutic agents. For example, vaccines have been developed using chimpanzee adenoviruses which have little to no seroprevalence in humans. However, it appears a significant percentage of some populations may still harbor some immunity to chimpanzee adenoviruses, as observed in a cohort from China (10).

Most attempts to develop adenoviruses with low seroprevalence have focused on those derived from species B or D, due to their comparative rarity (5, 9, 11). The most clinically advanced of these are HAdV-D26 and Enadenotucirev (formerly ColoAd1) (12). Enadenotucirev was developed by evolution of a panel of different adenovirus strains to select for recombinants with rapid replication in tumor cells. The resultant recombinant was predominantly HAdV-B11 with some elements of HAdV-B3 and has progressed into clinical trials as a novel cancer therapeutic (13, 14). HAdV-D26 is a replication-deficient vector and the basis of the Ad26.ZEBOV vaccine against Ebola virus that is currently under evaluation in the PREVAIL and PREVAC studies (15, 16).

Many species D adenoviruses have previously been evaluated for their potential as vaccines, gene therapies, and oncolytic viruses (1, 5, 9, 11). One with particularly low seroprevalence rates is HAdV-D49. In a cohort of 100 Belgian individuals only 2% had HAdV-D49-positive sera, whereas no preexisting immunity against HAdV-D49 was detected in 103 Scottish patients (8, 17). Prevalence is somewhat higher in sub-Saharan Africa with 22% of 200 patients presenting neutralizing antibodies, highlighting significant geographical variation in seroprevalence (9).

HAdV-D49 was first isolated from the feces of a human with no observed disease and later from Dutch patients (18, 19). It was then isolated from nosocomial epidemic keratoconjunctivitis infections (20, 21), but it is most associated with patients who are immunocompromised due to HIV infection (22). A study of adenovirus infections in patients from the United Kingdom and the Netherlands found 11 instances of HAdV-D49 infection in 183 HIV-positive patients (6% HAdV-D49 positive) compared to just two instances in 2,301 tested healthy patients (0.09% HAdV-D49 positive) (19).

Previous studies suggest that HAdV-D49 may be effective as a vaccine vector. A vaccine vector based on HAdV-D49 has been evaluated previously for its ability to protect against simian immunodeficiency virus (SIV) challenge. This vector induced strong anti-SIVGag CD8^+^-mediated immunity to SIV, greater than the comparable HAdV-C5-based vector (23). Another study sought to exploit HAdV-D49 a gene therapy to reduce excessive smooth muscle cell proliferation in vascular conduits following bypass grafting. This study demonstrated that HAdV-D49 was efficient at infecting endothelial cells and vascular smooth muscle cells, even after short exposure times (8). Studies in CAR (coxsackievirus and adenovirus receptor), CD46, and α2-3-linked sialic acid-expressing cells have previously suggested that HAdV-D49 may engage CD46 as a cellular receptor, although the effects observed were small (23).

Despite these studies and the development of HAdV-D49 as a therapeutic agent, there remains little information surrounding the basic biology of HAdV-D49 and its means of cellular engagement. Here, we investigate the tropism of HAdV-D49, focusing on the fiber knob protein as the major mediator of cellular attachment and evaluate the potential utility of a pseudotyped HAdV-C5/D49K vector to infect a range of cancer cell lines.

## RESULTS AND DISCUSSION

### HAdV-C5/D49K is not dependent on any known adenovirus receptor for cell entry.

**FIG 1:**
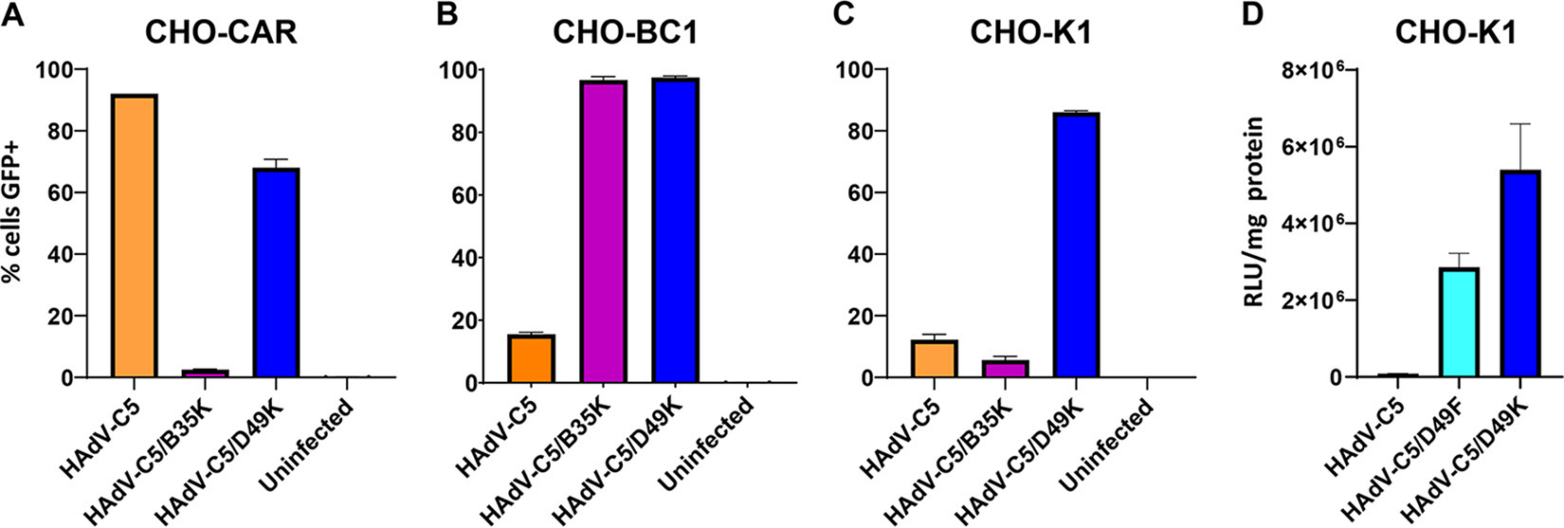
HAdV-C5/D49K infection is not dependent upon CAR or CD46. Transduction assays were performed in Chinese hamster ovary (CHO) cells stably expressing CAR (A), CHO-BC1 cells stably expressing human CD46 isoform BC1 (B), or CHO-K1 cells, which do not express any known adenovirus fiber knob receptors (C and D). Cells were infected with 5,000 viral particles per cell of replication-deficient HAdV-C5, HAdV-C5/B35K, or HAdV-C5/49K expressing a GFP transgene (A to C) or HAdV-C5, HAdV-C5/49K, or HAdV-C5/49F expressing luciferase (D). *n* = 3; error is expressed ± the standard deviations (SD).

To investigate the receptor usage of human adenovirus type 49 fiber knob protein, we generated a replication-incompetent HAdV-C5 vector pseudotyped with the fiber knob protein of HAdV-D49 (HAdV-C5/D49K), expressing either green fluorescent protein (GFP) or luciferase as transgenes. We also produced a replication-deficient HAdV-C5-based pseudotyped vector with the whole fiber protein, including both the fiber shaft and the fiber knob of HAdV-D49, expressing luciferase (HAdV-C5/D49F). This pseudotyping approach is a well-established means to investigate the fiber knob in the context of a well-understood, replication-incompetent virus (1, 24). Using these pseudotyped vectors, we performed transduction assays in CHO cells expressing common adenovirus receptors (Fig. 1). CHO-K1 cells do not express any known adenovirus receptor, while CHO-CAR cells express the HAdV-C5 receptor, coxsackievirus and adenovirus receptor (CAR), and CHO-BC1 cells express the BC1 isoform of CD46, the major receptor for species BI adenovirus, which includes HAdV-B35.

The ability of HAdV-C5/D49K to transduce cell lines was compared to similar GFP-expressing replication-incompetent vectors HAdV-C5 and HAdV-C5/B35K, which engage CAR and CD46 as receptors, respectively (24–26). HAdV-C5/D35K was unable to transduce CHO-CAR, due to the lack of CD46, while HAdV-C5 transduced CHO-CAR cells efficiently due to the high levels of CAR expressed. HAdV-C5/D49K transduced CHO-CAR cells efficiently, but slightly less so (by ∼20%) compared to HAdV-C5 (Fig. 1A). In CHO-BC1 cells, transduction by HAdV-C5 was inefficient, due to the absence of CAR, while HAdV-C5/B35 transduced these cells with almost 100% efficiency, due to the presence of high-affinity HAdV-B35 receptor, CD46. HAdV-C5/D49K demonstrated a similar ability to HAdV-C5-B35K to transduce CHO-BC1 cells (Fig. 1B). In CHO-K1 cells neither HAdV-C5 nor HAdV-C5/B35K were able to efficiently transduce the cells due to the absence of known adenovirus cell surface receptors. HAdV-C5/D49K, however, was able to transduce these cells efficiently, indicating that HAdV-D49K is able to infect cells efficiently and independently of CAR or CD46 (Fig. 1C), thus indicating HAdVC5/D49K engages an alternative cellular receptor. Interestingly, we also observed, in this and later experiments, that HAdV-C5/D49K was less efficient at infecting CHO-CAR cells (Fig. 1A) compared to non-CAR-expressing CHO cell types (Fig. 1B and C). These data indicate that the presence of CAR may actively reduce the efficiency of transduction of HAdVC5/D49K compared to levels of transduction in the absence of CAR in the same cell line background.

At 385 amino acids in length, the native HAdV-D49 protein is significantly shorter than the equivalent HAdV-C5 fiber protein, which is 581 amino acids in length. This manifests as a naturally shorter and less flexible fiber shaft in HAdV-D49 compared to that of HAdV-C5. This shortened fiber shaft length may impact upon viral infectivity, resulting in trapping of adenoviral particles within late endosomes due to the decreased endosomolytic activity of shorter shafted adenoviral particles (27), reviewed elsewhere (28). To assess the impact of pseudotyping the entire short fiber protein from HAdV-D49 on viral infectivity, we performed similar transduction assays using CHO-K1 cells. Consistent with engaging an alternative receptor on CHO-K1 cells, the HAdV-C5/D49F whole-fiber pseudotyped vector efficiently transduced CHO-K1 cells, where HAdV-C5 was unable. Also consistent with previous observations of shorter-shafted HAdVs potentially displaying reduced infectivity due to altered or less efficient intracellular trafficking postentry, the HAdV-C5/D49F was less efficient than the “knob-only” pseudotype HAdV-C5/D49K at infecting CHO-K1 cells (Fig. 1D).

**FIG 2:**
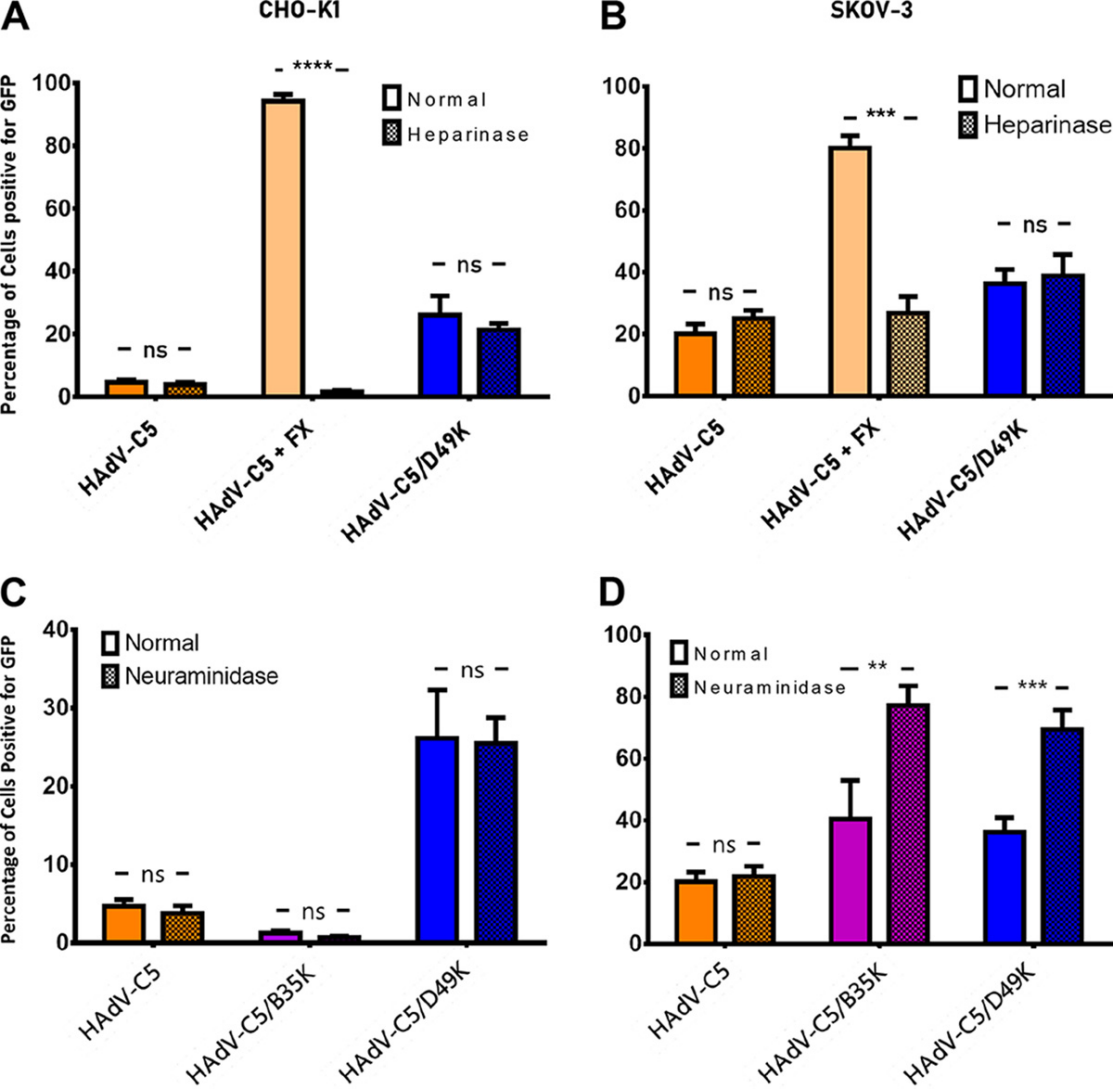
HAdV-C5/49K transduction is not dependent upon HSPGs, sialic acid bearing glycans or Desmoglein 2 (DSG2). Transduction assays were performed in CHO-K1 (A) or SKOV-3 (B) cells with or without heparinase pretreatment. As a positive control, HAdV-C5 assays were performed also in the presence of 10 μg/ml of FX. Transduction assays were performed with the indicated viral vectors in CHO-K1 (C) or SKOV-3 (D) cells that had been pretreated with neuraminidase to cleave cell surface sialic acid. Cells were infected with 5,000 viral particles per cell of replication-deficient HAdV-C5, HAdV-C5/B35K, or HAdV-C5/49K expressing a GFP transgene. *n* = 3; error is expressed ± the SD. *, *P* < 0.05; **, *P* < 0.01; ***, *P* < 0.005; ****, *P* < 0.001 (based on a nonparametric Mann-Whitney test).

We performed similar transduction assays using CHO-K1 and SKOV-3 ovarian cancer cells with or without pretreatment with either heparinase or neuraminidase to determine the ability of HAdVC5/D49K to bind heparan sulfate proteoglycans (HSPGs) or sialic acid, respectively, to mediate cellular infection (Fig. 2). As a positive control for heparinase activity we compared HAdV-C5/D49K infectivity to that of HAdV-C5 in the presence or absence of coagulation factor X (FX), a blood coagulation factor which can facilitate infection of some adenovirus by binding to the viral hexon and cellular HSPGs (29). We observed in CHO-K1 and SKOV-3 cells that transduction levels of HAdV-C5 alone were poor (Fig. 2A and B) but were significantly enhanced by the presence of FX, enabling cell entry through cellular HSPGs (30–32). Treatment with heparinase to cleave HSPGs reduced transduction efficiency to that of HAdV-C5 alone. HAdV-C5/D49K transduction efficiency was unaffected by treatment with heparinase (Fig. 2A and B), indicating that HAdV-D49 is unlikely to utilize HSPGs for cell entry.

Treatment with neuraminidase to remove cellular sialic acid did not alter the ability of any of the viruses to transduce CHO-K1 cells (Fig. 2C), as we previously demonstrated to show the involvement of sialic acid in HAdV-C5/D26K infection (24). In SKOV-3 cells, removal of sialic acid actually enhanced the transduction mediated by HAdV-C5/D49K and HAdV-C5/B35K, an effect which we have previously observed by neuraminidase treatment in SKOV-3 cells (Fig. 2D) (24, 33, 34). This effect could be a result of the removal of sialic acid enhancing nonspecific charge-based interactions between the cell surface and viral capsid. Regardless, these data do not support a role for sialic acid in HAdV-C5/D49K cell infection.

The transduction affinity of HAdV-C5/D49K in the experiments in Fig. 2 was noticeably weaker than in the CHO cell experiments (Fig. 1). This is due to the methodology used in each experiment. In the transduction experiments (Fig. 1), cells were incubated with virus at 37°C for 3 h. For studies evaluating the role of sialic acid and HSPGs, cells were pretreated with enzyme for 1 h at 37°C. The virus was then incubated with cells on ice for 1 h after enzymatic digestion to prevent repair and reconstitution of the cleaved heparin/sialic acid. This incubation on ice (and for a shorter period of time) likely decreases viral internalization during the absorption step, seemingly more profoundly for HAdV-C5/D49K than for the HAdV-C5, suggesting weaker binding at the cell surface or a comparatively low frequency of cell surface receptor.

**FIG 3:**
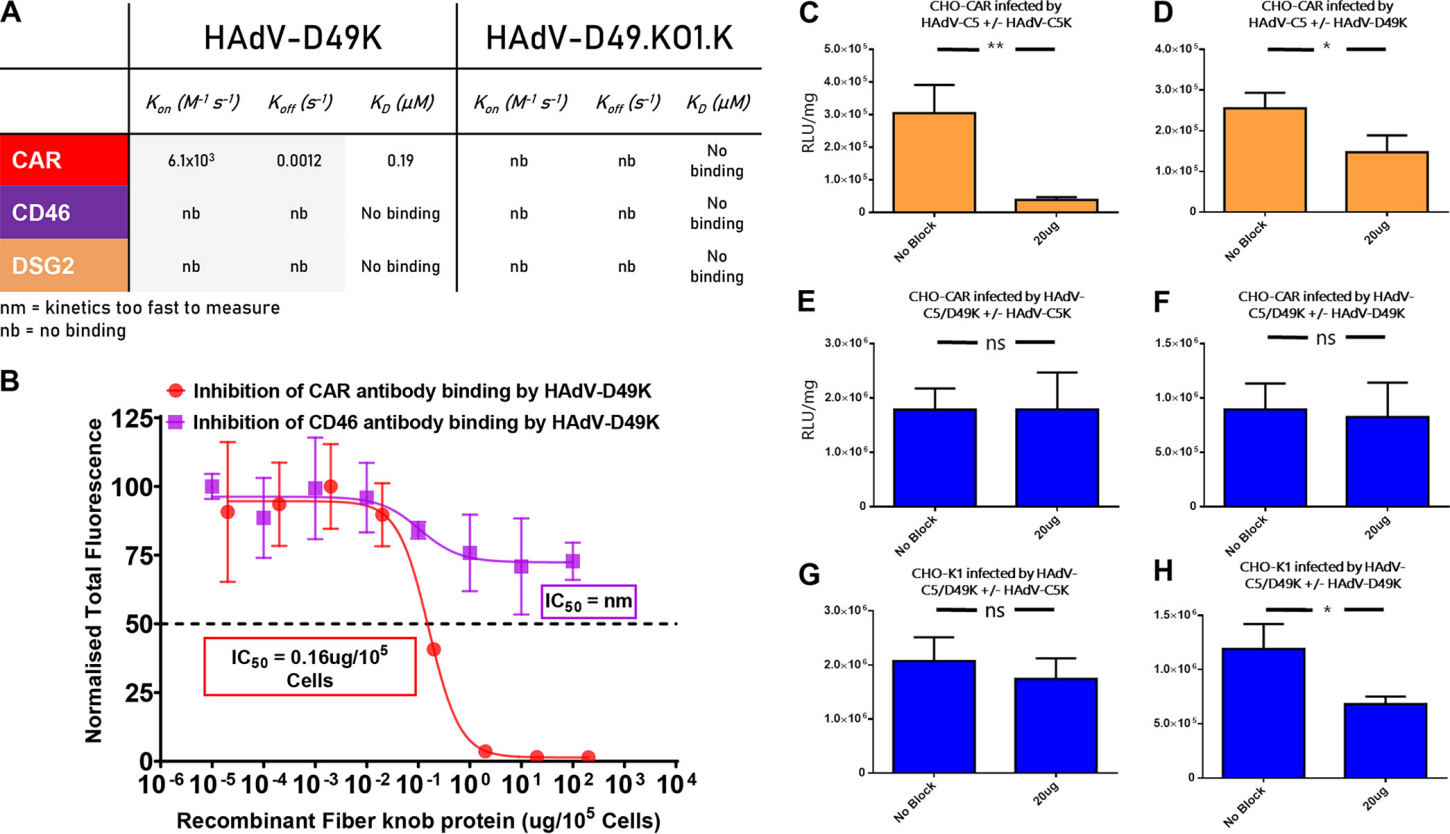
HAdV-D49K interacts with CAR but is not dependent upon it as an entry receptor. (A) SPR was used to detect potential interactions between HAdV-D49K or HAdV-D49.KO1.K and CAR, CD46, and DSG2. (B) Antibody binding inhibition assays were used to assess the ability of recombinant HAdV-D49K to inhibit antibody binding to CHO-CAR or CHO-BC1 cells. (C and D) Blocking of HAdV-C5-mediated transduction was studied by preincubation of CHO-CAR cells with HAdV-C5K (C) or HAdV-D49K (D). (E and F) Blocking of HAdV-C5/D49K-mediated transduction was studied by preincubation of CHO-CAR cells with HAdV-C5K (E) or HAdV-D49K (F). (G and H) Blocking of HAdV-C5/D49K-mediated transduction was studied by preincubation of CHO-K1 cells with HAdV-C5K (G) or HAdV-D49K (H). Cells were infected with 5,000 viral particles per cell of replication-deficient HAdV-C5 or HAdV-C5/D49K expressing a luciferase transgene, with or without blockade by 20 μg of recombinant HAdV-C5 or HAdV-D49 fiber knob protein. *n* = 3; error is expressed ± the SD. *, *P* < 0.05; **, *P* < 0.01 (based on a nonparametric Mann-Whitney test).

Desmoglein 2 (DSG2) is the other remaining well-established adenovirus receptor. DSG2 is described to interact with species BII adenovirus, including HAdV-B3K, via a low-affinity, avidity-dependent mechanism (35). We investigated whether HAdV-D49K might also interact with DSG2 by utilizing surface plasmon resonance (SPR), which we have previously used to establish a 66.9 μM affinity between DSG2 and HAdV-D3K (36). HAdV-D49K had no detectable affinity for DSG2 (Fig. 3A), an unsurprising finding since DSG2 has never been observed as a receptor for any adenovirus outside species B.

### HAdV-D49 fiber knob can interact with CAR but does not require it for cell entry.

We also used SPR to further probe the binding affinity of HAdV-D49K, and a mutant version, HAdV-D49.KO1.K, for CD46 and CAR. This HAdV-D49.KO1.K mutant, harbors the KO1 mutations S408E and P409A in the fiber knob AB loop, previously shown to ablate CAR binding in HAdV-C5K (37). The structure of the HAdV-D49.KO1.K fiber knob is also presented (Table 1, PDB 6QPO) (38). As predicted, we did not observe binding between either fiber knob protein and CD46. However, we did observe HAdV-D49K binding to CAR with a detectable 0.19 μM affinity which was ablated by the KO1 mutation (Fig. 3A).

**TABLE 1:** Single crystal diffraction data collection statistics for fiber knob crystal structures determined in this study

Parameter	PDB entry		
	6STU (HAdV-D30K)	6QPN (HAdV-D49K)	6QPO (HAdV-D49K.KO1)
Data collection statistics			
Diamond beamline	I03	I03	I03
Date	04/18/2019	02/27/2017	02/27/2017
Wavelength	0.95372	0.97628	0.97628
			
Crystal data			
Crystallization conditions	0.1 M SPG, 25% (wt/vol) PEG 1500	0.1 M MMT^*a*^ (pH 8.0), 25% (wt/vol) PEG 1500	0.1 M MMT (pH 8.0), 25% (wt/vol) PEG 1500
pH	6.0	8.0	8.0
Unit cell dimensions			
*a*, *b*, *c* (Å)	63.35, 87.36, 217.86	106.83, 56.28, 115.70	105.17, 55.99, 116.03
α, β, γ (°)	90, 90, 90	90.00, 112.95, 90.00	90.0, 112.47, 90.0
Space group	P 2_1_ 2_1_ 2_1_	P 1 2 1	P 1 2 1
Resolution (Å)	2.39–54.76	2.74–106.54	2.45–57.07
Outer shell	2.39–2.45	2.74–2.81	2.45–2.51
*R*_merge_ (%)	0.116 (1.376)	8.2 (163.3)	9.7 (127.4)
*R*_meas_ (%)	0.134 (1.583)	9.7 (191.0)	11.4 (149.9)
CC1/2	0.998 (0.623)	0.981 (0.400)	0.994 (0.491)
*I*/σ〈*I*〉	11.2 (1.4)	6.8 (1.0)	9.2 (1.2)
Completeness (%)	100.0 (100.0)	99.1 (98.9)	99.3 (99.3)
Multiplicity	7.5 (7.8)	3.7 (3.7)	3.7 (3.7)
Total measurements	365,880 (28,110)	122,219 (9,179)	168,773 (12,231)
Unique reflections	48,897 (3,589)	33,350 (2,461)	45,958 (3,349)
Wilson B-factor (Å^2^)	48.7	68.9	59.9
			
Refinement statistics			
No. of refined atoms	9,623	9,083	9,356
No. of *R*_work_ reflections	46,478	31,740	43,733
No. of *R*_free_ reflections	2,348	1,609	2,225
*R*_work_/*R*_free_ (%)	21.4/26.3	21.1/25.9	19.4/23.5
RMSD			
Bond length (Å)	0.009	0.009	0.012
Bond angle (°)	1.767	1.688	1.928
Coordinate error	0.288	0.369	0.264
Mean B value (Å^2^)	60.2	94.3	80.5
			
Ramachandran statistics (F/A/O)^*b*^			
No.	1,090/107/10	1,004/104/35	1,065/87/36
%	90.3/8.9/0.8	87.8/9.1/3.1	89.7/7.3/3.0

a MMT, malic acid plus MES plus Tris.

b F/A/O, favored/allowed/outliers.

We performed 50% inhibitory concentration (IC_50_) binding studies using recombinant HAdV-D49K protein on CHO-CAR and CHO-BC1 cells and assessed the ability of the recombinant fiber knob protein to inhibit the binding of anti-CAR or CD46 antibodies, respectively (Fig. 3B). HAdV-D49K was able to block anti-CAR antibody binding to CHO-CAR cells in a dose-dependent manner (IC_50_ = 0.16 μg/10^5^ cells, Fig. 3B). However, no IC_50_ could be derived by using HAdV-D49K to block CD46 on CHO-BC1 cells, where HAdV-D49K was unable to achieve >20% inhibition of antibody binding, suggesting weak or incidental CD46 interactions (Fig. 3B). Therefore, these data support the findings from SPR and transduction experiments that HAdV-D49K may bind CAR with low affinity but does not bind CD46.

Our earlier findings indicated that HAdV-C5/D49K is not dependent upon CAR for cell entry (Fig. 1); however, our *in vitro* biological inhibition and SPR assays demonstrate CAR binding affinity (Fig. 3A and B). We further investigated this finding by performing transduction blocking experiments using the CAR engaging HAdV-C5 and HAdV-C5/D49K with recombinant fiber knob protein of each virus in CHO-CAR and CHO-K1 cells. As predicted, preincubation of CHO-CAR cells with recombinant HAdV-C5K efficiently inhibited HAdV-C5 infection (Fig. 3C), while blocking with HAdV-D49K inhibited infection by HAdV-C5 by approximately 50% (Fig. 3D). Infecting CHO-CAR cells with HAdV-C5/D49K pseudotype and attempting to block using HAdV-C5K (Fig. 3E) or HAdV-D49K (Fig. 3F) did not significantly inhibit transduction efficiency. Finally, infection of CHO-K1 cells by HAdV-C5/D49K and blocking with HAdV-C5K did not significantly inhibit transduction efficiency (Fig. 3G), while blocking with HAdV-D49K reduced transduction efficiency by approximately 50% (Fig. 3H).

These data confirm that HAdV-D49K is capable of binding to CAR, albeit at ∼1,000-fold-lower affinity than HAdV-C5 (36), but in a manner able to inhibit HAdV-C5 binding. These data confirm HAdV-C5/D49K is capable of entering cells though a non-CAR- mediated pathway, since HAdV-C5K cannot inhibit HAdV-C5/D49K transduction in CHO-CAR cells (Fig. 3E). Interestingly, HAdV-C5/D49K was able to inhibit its own viral infection only in the absence of CAR (Fig. 3H).

One potential explanation for this activity is that the unknown alternative receptor to CAR has a lower affinity for HAdV-D49K than CAR. Therefore, in the presence of CAR the recombinant fiber knob would be sequestered on the higher-affinity CAR receptor, leaving the alternative receptor free to interact with the virus. A low-affinity receptor would also likely depend upon avidity and so might not be observed with single trimers of HAdV-D49K; a similar effect has previously been observed with HAdV-B3K and DSG2 (39) and CD46 (40). This is supported by the observation that HAdV-D49K cannot transduce cells as efficiently when incubated on ice in the absence of CAR, whereas HAdV-C5 and HAdV-C5/B35K, which form high-affinity receptor interactions, are unencumbered (Fig. 2).

### HAdV-D49K may bind cells through a charge-dependent mechanism.

**FIG 4:**
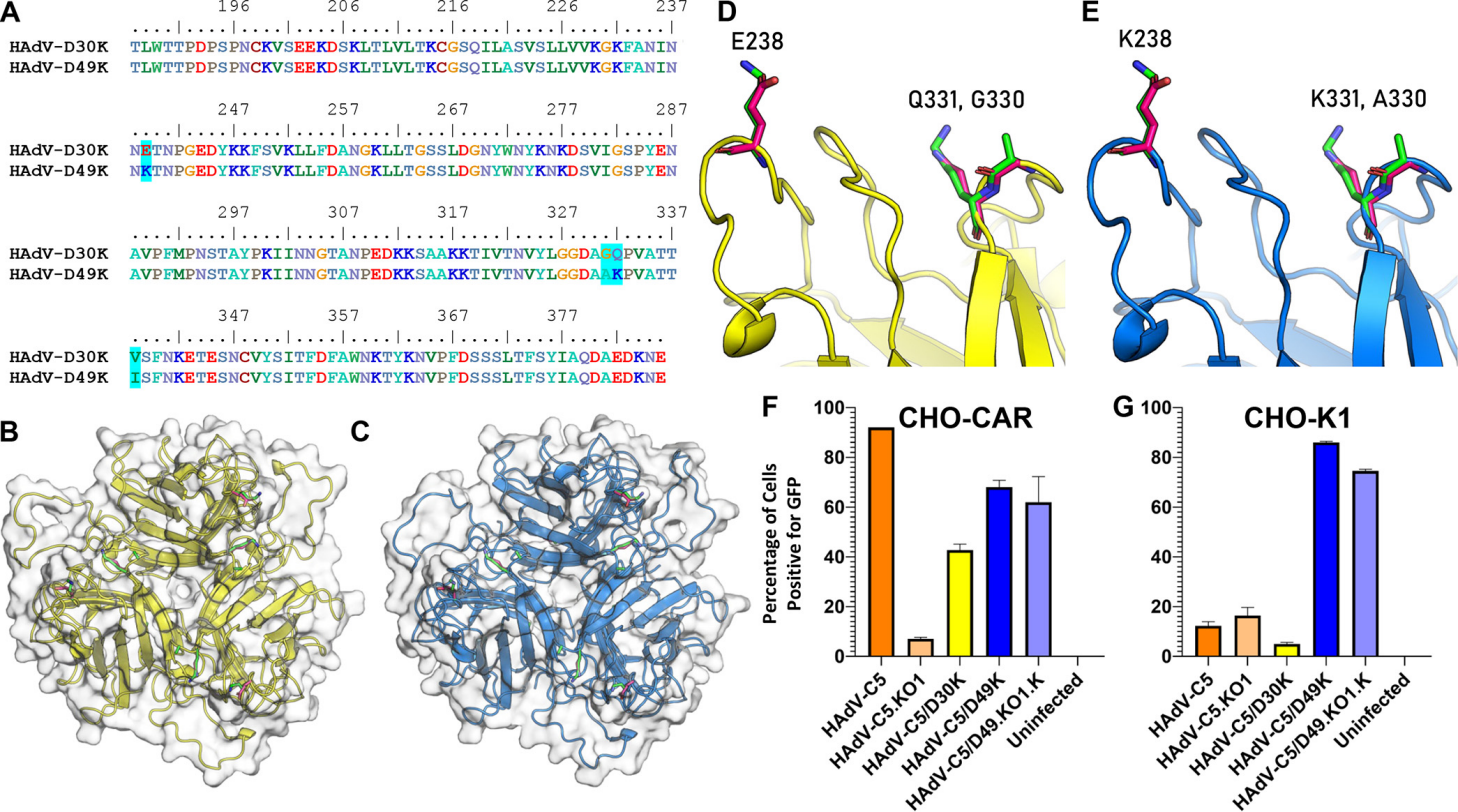
HAdV-D49K differs from HAdV-D30K in only three surface-exposed amino acids but demonstrates radically altered cellular tropism. (A) Clustal Ω sequence alignment (numbering is based on the whole fiber sequence) of HAdV-D30K and HAdV-D49K. (B and C) Viewed from the apex down the 3-fold axis, as if toward the viral capsid, the crystal structures of HAdV-D30K (B) and HAdV-D49K (C) reveal that three of these residues are surface exposed. These residues can be seen projecting into the solvent from loops on the apex of HAdV-D30K (D) and HAdV-D49K (E). Residue numbers and names correspond to the fiber knob protein depicted in that frame. Sticks representing residues belonging to HAdV-D30K and HAdV-D49K are seen in pink and green, respectively. (F and G) Transduction assays were performed to assess tropism of HAdV-C5/D30K, HAdV-C5/D49K, and HAdV-C5/D49.KO1.K in CHO-CAR cells (F) and CHO-K1 cells (G). Cells were infected with 5,000 viral particles per cell of replication-deficient HAdV-C5 or HAdV-C5/D49K expressing a luciferase transgene, with or without blockade by 20 μg of recombinant HAdV-C5 or HAdV-D49 fiber knob protein.

To investigate other closely related HAdV with homologous fiber knob proteins, we performed a BLASTp search using the HAdV-D49K amino acid sequence. This search revealed that the HAdV-D30K protein is highly homologous to HAdV-D49K, differing in just 4 amino acid residues (Fig. 4A).

We solved the crystal structures of HAdV-D30K (Fig. 4B) and HAdV-D49K (Fig. 4C). Diffraction data collection statistics for these structures are provided in Table 1. We demonstrate that structurally, HAdV-D30K and HAdV-D49K are highly homologous (root mean square deviation [RMSD] = 0.292 Å^2^). Residue 338 is not surface exposed on either fiber knob protein and is likely to be functionally homologous (HAdV-D49 = isoleucine-338, HAdV-D30 = valine-338). However, the remaining three residue differences, E238K, G330A, and Q331K (HAdV-D30K → D49K) are surface exposed at the apex of each fiber knob monomer (Fig. 4D and E). The E238K and Q331K substitutions have opposing charges.

We investigated the transduction efficiency of HAdV-C5/D30K compared to HAdV-C5/D49K, HAdV-C5, HAdV-C5.KO1, and HAdV-C5/D49.KO1.K in CHO-CAR (Fig. 4F) and CHO-K1 (Fig. 4G) cells. HAdV-C5 infected CHO-CAR cells efficiently, while the CAR-binding ablated KO1 mutant did not, and HAdV-C5/49K and the corresponding mutant HAdV-C5/49KO1.K infected CHO-CAR cells with similar efficiency, as observed for HAdV-C5/D49K in Fig. 1A. HAdV-C5/D30K infected CHO-CAR cells with approximately 40% efficiency (Fig. 4F). In CHO-K1 cells, HAdV-C5/D49K and the KO1 mutant were the only viruses that achieved efficient transduction. Surprisingly, given the high homology to HAdV-C5/D49K, the HAdV-C5/D30K pseudotype was inefficient in transducing CHO-K1 cells (<5% GFP^+^, Fig. 4G).

**FIG 5:**
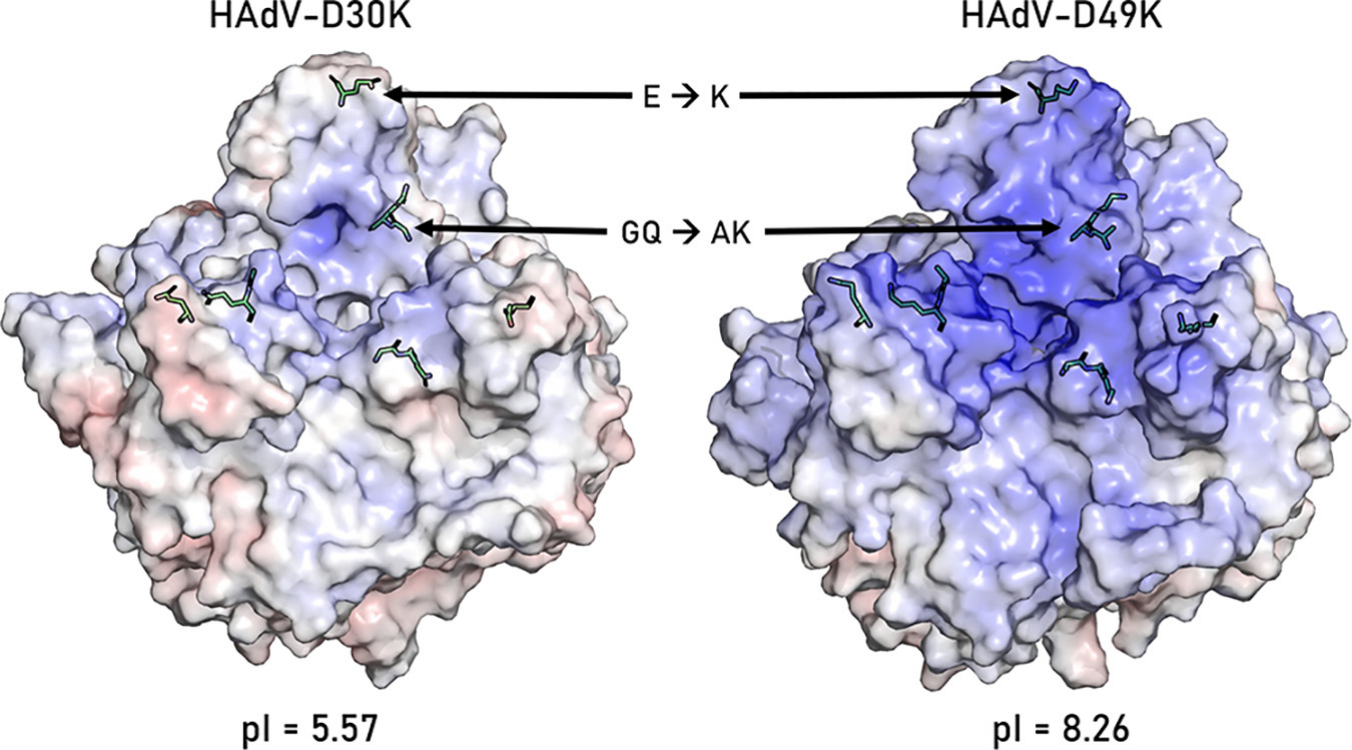
The residue differences between HAdV-D30K and HAdV-D49K affect the surface electrostatic potential of the fiber knob. The calculated pIs of HAdV-D30K and HAdV-D49K differ as a result of the residue changes, which are shown as green sticks as they occur in that fiber knob protein. The calculated electrostatic surface potential at pH 7.35 is projected on a ramp from –10 mV to +10 mV (red to blue). HAdV-D49K has much more basic potential around the apex, where the residue substitutions are located.

This profound difference in transduction efficiency between HAdV-C5/D30K and HAdV-C5/D49K must be dependent upon the three surface-exposed amino acid differences. We investigated the effect of the opposing charges at residue substitutions 238 and 331 (Fig. 4A to E) by modeling the electrostatic surface potential of the two fiber knob proteins, based on our crystal structures (Fig. 5). The surface potential maps reveal that while structural homology was high, they present radically different electrostatic surface potential distributions. HAdV-D30K is significantly more acidic (pI = 5.57) than HAdV-D49K (pI = 8.26) (Fig. 5).

Thus, it seems probable that the interaction with the unknown cell surface receptor requires basic electrostatic potential. This is commensurate with the previous inference that its receptor is likely to be low affinity, since electrostatic interfaces are often observed to be less stable than their ionic counterparts. It is possible that the electrostatic potential differences explain the reduced transduction affinity observed in HAdV-C5/D30K compared to HAdV-C5/D49K in CHO-CAR cells. Should the strong charge on HAdV-D49K be opposed to that on the surface of CAR, this could enhance the interaction stability and therefore the overall virus affinity. It seems unlikely that the residue substitutions themselves would strongly influence CAR affinity since they occur at the apex of the fiber knob, an area that is not critical for the CAR interface (1).

### HAdV-C5/D49K is able to efficiently infect a large range of cancer cell lines.

Given that HAdV-C5/D49K infects cells independently of known adenovirus receptors, we hypothesized that it may form the basis of an efficient vector for cancer virotherapy applications. We therefore compared its transduction efficiency to that of HAdV-C5 in panels of pancreatic, breast, esophageal, colorectal, ovarian, and lung cancer cell lines (Table 2).

**TABLE 2:** Comparison of transduction of various cancer cell lines by HAdV-C5 and HAdV-C5/D49K

Cancer type	Cell line	Infection level (RLU/mg total protein)		Change in infectivity (fold change)^*a*^	*P*^*b*^
		HAdV-C5	HAdV-C5/D49K		
Pancreatic adenocarcinoma, derived from acitic metastasis	ASPC1	3.9E+04	1.2E+06	30.7	**
Pancreatic adenocarcinoma	BXPC3	1.2E+04	2.5E+06	210.9	**
	Panc10	1.0E+05	7.2E+06	69.2	****
Pancreatic adenocarcinoma, derived from splenic metastasis	SW1990	9.6E+04	1.1E+06	11.3	****
Pancreatic adenocarcinoma	MIA PaCa2	3.4E+05	1.4E+06	4.2	**
Pancreatic adenocarcinoma, derived from liver metastasis	Suit2	6.0E+04	1.3E+06	22.2	*
Pancreatic ductal adenocarcinoma	PANC 0403	1.2E+04	4.0E+05	33.0	*
Pancreatic ductal adenocarcinoma, derived from liver metastasis	CFPAC-1	1.8E+04	1.1E+06	62.0	***
Pancreatic ductal adenocarcinoma	PT45	3.9E+05	2.0E+06	5.1	**
Breast carcinoma	BT-20	1.8E+03	8.6E+05	481.2	*
Breast ductal carcinoma	BT-474	6.5E+03	1.4E+05	21.8	*
Breast adenocarcinoma, derived from brain metastasis	MDA-MB-361	2.2E+05	1.5E+06	6.7	**
Breast adenocarcinoma, derived from plural effusion metastasis	MDA-MB-231	3.3E+04	1.5E+05	4.6	*
	MCF7	1.3E+04	6.6E+06	497.3	***
Large lunge cell carcinoma	NCI-H460	2.1E+05	3.9E+06	18.8	*
Lung carcinoma	A427	1.2E+07	7.2E+06	0.6	*
	A549	2.8E+06	6.9E+06	2.5	***
Esophageal squamous cell carcinoma	Kyse-30	6.3E+05	1.4E+07	22.4	***
Colorectal adenocarcinoma, Duke’s type C	DLD-1	6.0E+06	5.0E+06	0.8	ns
Ovarian adenocarcinoma	SKOV-3	9.1E+05	7.7E+06	8.5	**
Gastric tubular adenocarcinoma	MKN28	2.6E+05	2.0E+07	76.9	***
Gastric adenocarcinoma	MKN45	3.1E+04	2.2E+06	71.0	***

a The fold change was determined as the RLU/mg of HAdV-C5/D49K divided by the RLU/mg of HAdV-C5.

b Statistical significance (*P*), determined using a Student t test, is indicated as follows: *, *P* < 0.05; **, *P* < 0.01; ***, *P* < 0.001; ****, *P* < 0.0001; or ns, not significant.

In pancreatic cancer cell lines, HAdV-C5/D49K was consistently more efficient at cellular transduction than HAdV-C5. This improved activity ranged between 4.2× more efficient in MiaPaCa2 cells to 210.9× more efficient in BxPc3 cells. The most effectively transduced cell line was Panc10 cells, producing 7.2 × 10^6^ relative light units (RLU)/mg of fluorescence, compared to the least efficient at just 4.0 × 10^5^ RLU/mg in Panc0403 cells. This suggests that the large differences between HAdV-C5/D49K and HAdV-C5 transduction levels are likely due to the variability in the expression of CAR. A similarly broad range of different relative infection efficiencies was observed in the breast cancer cell lines studied. In MCF7 cells and BT20 cells, HAdV-C5/D49K was nearly 500-fold more efficient at transducing the cells due to these cells expressing low levels of CAR, with consequent poor levels of HAdV-C5-mediated transduction (Table 2).

DLD-1 colorectal cancer cells were efficiently transduced by both HAdV-C5 and HAdV-C5/D49K vectors at 5.0 × 10^6^ and 6.0 × 10^6^ RLU/mg, respectively. This is the only cell line where no significant difference in infectivity was observed. A427 lung carcinoma cells are the only line where HAdV-C5-mediated cellular transduction was more efficient than HAdV-C5/D49K (0.6×). While HAdV-C5/D49K transduced A427 cells very efficiently (7.2 × 10^6^ RLU/mg), HAdV-C5 achieved an unusually high transduction efficiency (1.2 × 10^7^ RLU/mg) (Table 2). Therefore, this result is not due to inefficient HAdV-C5/D49K transduction but to unusually efficient HAdV-C5 transduction.

### Conclusions.

Previous experiments using whole HAdV-D49 virus concluded that it utilizes CD46 as its primary cellular receptor (23). The data presented, generated using either purified HAdV-D49 fiber knob protein or a pseudotyped HAdV-C5/D49K vector, clearly demonstrate CD46 to be implausible as a receptor for HAdV-D49.

We demonstrate that the HAdV-D49 fiber knob has a weak affinity for CAR, although it is not dependent upon this interaction to mediate efficient cell entry, and in fact the presence of CAR may inhibit cellular transduction. In support of this, we show that a mutant vector, HAdV-C5/D49.KO1.K, containing mutations within the fiber knob domain which ablate CAR affinity, retained the ability to efficiently transduce cells in the absence of any detectable binding to CAR. Based on the low efficiency by which HAdV-C5/D49K transduced cells when absorbed on ice and the observation that HAdV-D49K is only capable of inhibiting HAdV-C5/D49K transduction in the absence of CAR, we tentatively suggest that the unknown receptor is likely to be bound with weak affinity and virus attachment may be avidity dependent.

Regardless of the mechanism of interaction, this study strongly suggests there is an as-yet-unknown adenovirus receptor or mechanism of cell entry which mediates efficient transduction of a broad range of cell lines. This is demonstrated by its ability to efficiently infect every cell line tested throughout this study. The weakest observed transduction was in Panc0403 cells, where it achieved 4.0 × 10^5^ RLU/mg of luminescence. Although this is not a particularly strong transduction efficiency, it is still significantly higher (33.0×, *P* < 0.05) than that of HAdV-C5. It is likely, therefore, that the HAdV-C5/D49K vector described here may be useful in biotechnology applications to efficiently express proteins in difficult-to-transduce cell lines.

HAdV-C5/D49K represents a highly efficient gene transfer vehicle that is not restricted by any known adenovirus tropism. It possesses a broad range of infectivity and has potential as both a laboratory reagent for the transient expression of transgenes and as a therapeutic vaccine or oncolytic virus. For oncolytic applications, it is likely that a further refinement, such as the introduction of mutations known to confer tumor selective replication, such as a dl24 mutation (41–43), or the use of tumor-specific promoters, such as hTERT (44) or Survivin (45), to drive transgene therapeutic expression selectively within tumor cells will be necessary to ensure tight tumor selectivity.

## MATERIALS AND METHODS

### GFP transduction assay.

Adherent cells were seeded into a Nunc delta surface 96-well cell culture plate (Thermo Fisher) at a density of 5 × 10^4^ cells/well in 200 μl of cell culture media and left to adhere overnight at 37°C in a 5% CO_2_ humidified atmosphere. The medium was removed, and cells were washed twice with 200 μl of phosphate-buffered saline (PBS). Virus was added at the desired concentration in 200 μl of serum-free RMPI 1640, followed by incubation for 3 h. The virus-containing medium was then removed and replaced with complete cell culture medium, and the cells were incubated for a further 45 h. The cell culture medium was then removed, and the cells were washed twice with 200 μl of PBS, trypsinized in 50 μl of 0.05% trypsin-EDTA (Gibco), and dissociated by pipetting. The trypsinized cells were transferred to a 96-well V-bottom plate (Thermo Fisher), neutralized with 100 μl of complete cell culture media, and pelleted by centrifugation at 1,200 rpm for 3 min. The supernatant was removed, and the cells were washed once in 200 μl of PBS and resuspended in 100 μl of 2% paraformaldehyde (PBS containing 2% [wt/vol] paraformaldehyde), followed by incubation at 4°C for 15 min. The cells were again pelleted, washed twice in 200 μl of PBS, and then resuspended in 200 μl of PBS prior to analysis by flow cytometry.

Samples were analyzed by flow cytometry on an Attune NxT (Thermo Fisher), and voltages were set prior to each experiment, for each cell type, using an uninfected cell population treated identically. Data were analyzed using FlowJo (v10; FlowJo, LLC), gating sequentially on singlets, cell population, and GFP-positive cells. The levels of infection were defined as the percentage of GFP-positive cells (% +ve) and/or the total fluorescence (TF), defined as the % +ve multiplied by the median fluorescent intensity (MFI) of the GFP-positive population. These measures are distinct in that % +ve describes the total proportion of cells infected, and TF describes the total efficiency of transgene delivery.

### Luciferase transduction assay.

Luciferase infectivity assays were performed using the luciferase assay system kit (Promega). Cells were seeded into a Nunc delta surface 96-well cell culture plate (Thermo Fisher) at a density of 2 × 10^4^ cells/well in 200 μl of cell culture media and left to adhere overnight at 37°C in a 5% CO_2_ humidified atmosphere. The medium was removed, and the cells were washed once with 200 μl of PBS. Luciferase transgene encoding replication-incompetent viruses were added to the wells at the required titer in 200 μl of serum-free RMPI 1640, followed by incubation for 3 h. The virus-containing medium was then removed and replaced with complete cell culture medium, and the cells were incubated for a further 45 h. The cell culture medium was then removed, and the cells were washed twice with 200 μl of PBS and then lysed in 100 μl of cell culture lysis buffer (part of the Promega kit) diluted to 1× in ddH_2_O. The plate was then frozen at −80˚C.

After thaw, 10 μl of lysate from the cell culture plate mixed was then transferred to a white Nunc 96-microwell plate (Thermo Fisher), and 100 μl of luciferase assay reagent (Promega kit) was added. The luciferase activity was then measured as RLU by a plate reader (Clariostar; BMG Labtech). The total protein concentration was determined in the lysate by using a Pierce BCA protein assay kit (Thermo Fisher) according to the manufacturer’s protocol, and the absorbance was measured on an iMark microplate absorbance reader (Bio-Rad).

The relative virus infection was determined by normalizing the measured luciferase intensity to the total protein concentration (the RLU was divided by protein concentration). This gave a final infectivity readout in RLU/mg of protein.

### Blocking of virus infection with recombinant fiber knob protein.

This assay was also performed using the luciferase assay system kit (Promega). Cells were seeded into a Nunc delta surface 96-well cell culture plate (Thermo Fisher) at a density of 2 × 10^4^ cells/well in 200 μl of cell culture media and left to adhere overnight at 37°C in a 5% CO_2_ humidified atmosphere. The medium was removed, the cells were washed twice with 200 μl of cold PBS, and the plate was cooled on ice. Then, 20 pg/cell of recombinant adenovirus fiber knob was added to each well in 200 μl of cold PBS, followed by incubation on ice in a 4°C cold room for 1 h. The medium was then removed, and luciferase transgene encoding replication- incompetent viruses were added to the necessary wells at the required titer in 200 μl of cold serum-free RPMI 1640, followed by incubation on ice in a 4°C cold room for 1 h. The virus-containing medium was then removed and replaced with complete cell culture medium, and the cells were incubated for a further 45 h under normal cell culture conditions. From this point forward, the assay is identical to the GFP and luciferase transduction assays.

### Heparinase and neuraminidase transduction assays.

Cells were seeded at a density of 5 × 10^4^ cells/well in a flat-bottom 96-well cell culture plate and incubated overnight at 37°C to adhere. Cells were washed twice with 200 μl of PBS. Then, 50 μl of neuraminidase (from Vibrio Cholera, Merk) at a concentration of 50 mU/ml or 50 μl of heparinase III (from Flavobacterium heparinum [Merck]) at a concentration of 1 U/ml was diluted in serum-free media, added to the appropriate wells, and incubated for 1 h at 37°C. Cells were cooled on ice and washed twice with 200 μl of PBS. GFP-expressing, replication-incompetent viruses were added to the appropriate wells at a concentration of 5,000 viral particles per cell in 100 μl of serum-free media at 4°C, followed by incubation on ice for 1 h. Serum-free medium alone was added to uninfected control wells. Cells were washed twice with 200 μl of cold PBS, and complete medium was added (Dulbecco modified Eagle medium, 10% fetal calf serum), followed by incubation for a further 48 h at 37°C. Cells were then trypsinized and transferred to a 96-well V-bottom plate, washed twice in 200 μl of PBS, and fixed in 2% paraformaldehyde containing PBS for 20 min before washing and resuspension in 200 μl of PBS.

Samples were analyzed by flow cytometry on an Attune NxT (Thermo Fisher), and voltages were set prior to each experiment, for each cell type, using an uninfected cell population treated identically. Data were analyzed using FlowJo, gating sequentially on singlets, cell population, and GFP-positive cells. The levels of transduction were defined as the percentage of GFP-positive cells (% +ve), and/or total fluorescence (TF), defined as the percentage of GFP-positive cells multiplied by the MFI of the GFP-positive population. These measures are distinct in that % +ve describes the total proportion of cells infected, and TF describes the total efficiency of transgene delivery.

### Surface plasmon resonance.

Surface plasmon resonance was performed, in triplicate, as previously described, using recombinant HAdV-D49K protein (36). Approximately 5,000 RU of recombinant human desmoglein-2 Fc chimera protein (R&D Systems, catalogue no. 947-DM-100) was amine coupled to a CM5 sensor chip at a slow flow rate of 10 μl/min to ensure uniform distribution on the chip surface.

### Competition inhibition assay.

Competition inhibition assays of antibody binding to cell surface receptors were performed as previously described (36).

### Generation of recombinant fiber knob proteins.

Recombinant fiber knob proteins used in transduction inhibition, antibody blocking, and crystallization experiments were produced as previous described (24, 36). Briefly, pQE-30 vectors containing the sequence of the relevant fiber knob protein, spanning from 13 amino acids preceding the TLW motif to the stop codon, were transformed into SG13009 *Escherichia coli* harboring the pREP-4 plasmid. Portions (1 liter) of these *E. coli* were grown to and optical density of 0.6, and protein expression was induced with a final concentration of 0.5 mM IPTG (isopropyl-β-d-thiogalactopyranoside). *E. coli* was harvested by centrifugation and resuspended in 50 ml of lysis buffer (50 mM Tris [pH 8.0], 300 mM NaCl, 1% [vol/vol] NP-40, 1 mg/ml lysozyme, 1 mM β-mercaptoethanol). Sample was then loaded onto a HisTrap FF Crude column and eluted by using imidazole. Fractions determined to contain protein of interest were then concentrated to <1 ml total volume and purified by size exclusion chromatography using a Superdex 200 10/300 GL Increase column.

### Fiber knob protein crystallization and structure determination by X-Ray crystallography.

HAdV-D49K, HAdV-D49.KO1.K, and HAdV-D30K were crystallized as previously described (24, 36). Both HAdV-D49K and HAdV-D49.KO1.K crystals formed in 0.1 M MMT–25% (wt/vol) PEG1500, whereas HAdV-D30K crystallized in 0.1 M SPG–25% (wt/vol) PEG 1500. All crystals formed in 2 to 7 days in sitting-drop format. The data collection statistics are described in Table 1, and the structures were solved by molecular replacement using PDB 6FJN.

### Calculation of electrostatic surface potentials and pIs.

Electrostatic surface potential and isoelectric points were calculated at pH 7.2 using the PDB2PQR Server (V 2.1.1) (46) as previous described (24).

### RMSD calculation, sequence alignment, and imaging of crystal structures.

Alignments were performed using the Clustal Omega multiple sequence alignment algorithm and visualized with BioEdit (47, 48). RMSD calculations were performed using the “align” command in PyMOL 2.0, which was also used to visualize protein structures (49).

### Data availability.

The following proteins have been deposited in the Protein Data Bank (PDB) under the indicated accession numbers: adenovirus 30 fiber knob protein (6STU), adenovirus species D serotype 49 fiber knob (6QPN), and adenovirus species D serotype 49 fiber knob KO1 mutant (6QPO).
